# Hereditary Polyneuropathies in the Era of Precision Medicine: Genetic Complexity and Emerging Strategies

**DOI:** 10.3390/genes17010056

**Published:** 2026-01-03

**Authors:** Maria Chrysostomaki, Despoina Chatzi, Stella Aikaterini Kyriakoudi, Soultana Meditskou, Maria Eleni Manthou, Sofia Gargani, Paschalis Theotokis, Iasonas Dermitzakis

**Affiliations:** Department of Histology-Embryology, School of Medicine, Aristotle University of Thessaloniki, 54124 Thessaloniki, Greece; chrysmaria@auth.gr (M.C.); chatzidc@auth.gr (D.C.); kstellaai@auth.gr (S.A.K.); sefthym@auth.gr (S.M.); mmanthou@auth.gr (M.E.M.); sgargani@bio.auth.gr (S.G.); ptheotokis@auth.gr (P.T.)

**Keywords:** hereditary polyneuropathies, peripheral nerve, genetics, Schwann cell, gene therapy, precision medicine

## Abstract

Hereditary polyneuropathies represent a genetically and clinically heterogeneous group of disorders affecting the peripheral nervous system, characterized by progressive motor, sensory, and autonomic impairment. Advances in molecular genetics have identified key causative genes, including *PMP22*, *MPZ*, *MFN2*, *TTR*, *EGR2*, and *CX32 (GJB1)*, which are implicated in Charcot–Marie–Tooth disease, Dejerine–Sottas syndrome, and related neuropathies. These conditions display substantial allelic and locus heterogeneity. Pathogenetically, mechanisms involve impaired myelin maintenance, disrupted axonal transport, mitochondrial dysfunction, and aberrant Schwann cell biology. Despite these insights, therapeutic options remain limited, and there is a pressing need to translate genetic findings into effective interventions. This review aims to provide a comprehensive synthesis of current knowledge compiling all known mutations resulting in hereditary polyneuropathies. In addition, it underscores the molecular pathomechanisms of hereditary polyneuropathies and evaluates emerging therapeutic strategies, including adeno-associated virus mediated RNA interference, CRISPR-based gene editing, antisense oligonucleotide therapy, and small-molecule modulators of axonal degeneration. Furthermore, the integration of precision diagnostics, such as next-generation sequencing and functional genomic approaches, is discussed in the context of personalized disease management. Collectively, this review underscores the need for patient-centered approaches in advancing care for individuals with hereditary polyneuropathies.

## 1. Introduction

Hereditary polyneuropathies (HPNs) are a diverse group of genetic disorders affecting the peripheral nervous system (PNS), leading to progressive motor and sensory deficits that significantly impact patients’ quality of life. With an estimated prevalence of approximately 1 in 2500 individuals for Charcot–Marie–Tooth disease (CMT), which is the most common type of HPN, these disorders collectively represent one of the most frequent forms of inherited neurological disease [1]. Apart from CMT, HPNs include a wide range of conditions, such as hereditary neuropathy with liability to pressure palsies (HNPP), Dejerine–Sottas syndrome (DSS), congenital hypomyelinating neuropathy (CHN), Roussy–Levy Syndrome (RLS), and hereditary transthyretin amyloidosis (hTTR), each displaying variable severity and clinical manifestations. The disease spectrum ranges from mild distal weakness and sensory loss to severe functional disability, often resulting in reduced mobility, decreased independence, and significant healthcare costs.

At the genetic level, hereditary polyneuropathies exhibit marked genetic heterogenenity. Different mutations in the same gene can lead to distinct clinical phenotypes, while a single neuropathy may arise from variants in multiple genes. This complexity reflects the diverse biological pathways underlying disease pathogenesis. A major subset of HPNs arises from defects in genes critical for myelin synthesis and maintenance. Myelin in the PNS is produced by Schwann cells, glial cells that ensheathe axons to enable rapid nerve conduction. Mutations in genes encoding essential myelin proteins, such as PMP22 and MPZ, or stabilization proteins like periaxin, disrupt this process and impair nerve function [2]. Beyond myelin-related defects, alterations in genes involved in Schwann cell survival and function (*EGR2*, *CX32*), mitochondrial dynamics (*MFN2*), or protein transport (*TTR*) further contribute to disease development [3].

The overlapping clinical features and extensive genetic diversity of HPNs pose substantial challenges for accurate diagnosis and effective treatment. Conventional diagnostic approaches often fail to pinpoint the underlying genetic cause, emphasizing the importance of comprehensive genetic testing and molecular characterization [4]. Therapeutic options remain largely supportive, highlighting the urgent need for novel, targeted interventions [5].

While several reviews have examined hereditary polyneuropathies, most have focused either on clinical classification or specific gene subsets. This review aims to provide an updated synthesis of the known causative mutations and molecular mechanisms underlying these disorders, while exploring key genes associated, highlighting the diversity of pathogenic pathways and framing them within the context of precision medicine. By integrating current genetic and mechanistic insights, this review seeks to enhance understanding of these debilitating conditions and support the development of targeted therapeutic interventions.

## 2. Genes Involved in HPNs

### 2.1. Peripheral Myelin Protein 22 Gene (PMP22)

#### 2.1.1. Overview

*PMP22* is a protein coding gene whose product plays a crucial role in the PNS, as it is involved in growth regulation and myelination [6]. Importantly, *PMP22* is a dosage-sensitive gene, and genomic rearrangements affecting this locus can lead to the generation of copy number variants. Variations in copy number, either duplications or deletions of specific genomic intervals, are associated with distinct genomic disorders [7]. Specifically, *PMP22* encodes an integral membrane glycoprotein, predominantly expressed in Schwann cells, where it constitutes a key component of the myelin sheath surrounding axons. This sheath overlays and insulates the axon, thereby increasing the speed of the electrical signals transmission along the nerve [8].

Its supporting role can be direct or indirect, exhibiting dual properties. During peripheral nerve development, PMP22 ensures that the myelin sheath is functional, stable and consequently that the axon is protected. Regarding its direct role, the protein is speculated to regulate differentiation of Schwann cells, which are known for their supportive role in maintaining axonal integrity. Remarkably, overexpression of *PMP22* decreases the proliferation of Schwann cells in human subjects, affected by CMT1 [9]. All *PMP22* mutations, including duplication, deletion, and point mutations, cause increased apoptosis in cultured Schwann cells, fibroblasts, and myelinating Schwann cells from mice and humans with CMT1A. However, this apoptosis is only noticeable in aged Schwann cells in vivo and appears to be milder in PMP22 deficient cells compared to Schwann cells with *PMP22* overexpression or toxic-gain-of-function point mutations [10]. Given these observations, it is comprehensible that myelin dysfunction may occur when the normal protein is absent, potentially resulting in HPNs. Certain mutations in *PMP22* gene are associated with both type 1A and type 1E of CMT, HNPP, and a subtype of DSS [11]. All genetic alterations in *PMP22* that cause PNPs are listed in Table 1.

#### 2.1.2. Charcot-Marie-Tooth Type 1 (CMT1)

CMT1 is the most common form of HPNs. Despite persistent research, the genetic background of the disease is still not fully understood, perhaps because of the many different causes [19]. Several mutations can lead to this condition, but the most prevalent is a 1.5 Mb tandem duplication of 17p11.2–p12 [6,13]. About 71% of CMT1A patients appear to have this variation [30], which results in overexpression of *PMP22* and is inherited as an autosomal dominant trait. Less frequently, the same phenotype may arise from point mutations of the same gene [24,27,30]. In these cases, CMT1A or CMT1E forms occur [11]. A Asp37Val substitution, occurring in the first extracellular domain, is associated with a peculiar form of CMT1A [15]. A known missense mutation is a C to T transition at nucleotide 402 in exon 4, resulting in a threonine (T) to methionine (M) substitution at codon 118 [18]. This mutation (T118M) is also caused by a CpG dinucleotide sequence change. Such DNA sites have a high mutation rate in human DNA after being methylated, due to the spontaneous deamination of the 5-methylcytosine [31]. Recent studies revealed certain mutations that are less frequent causes of CMT1. Those mutations include a G to A transition at nucleotide 178, leading to p. Glu60Lys amino acid change in exon 3, a 3215 deletion, which also includes exon 4 and p. His12Pro variations [19].

The pathophysiological pathway of this condition is quite complicated, and it is associated with two different mechanisms. *PMP22* overexpression dysregulates the expression of myelin proteins, which results in the suppression of enzymes that catalyse the production of cholesterol [17]. A lack of cholesterol production during the development of peripheral nerves is known to impact myelination, resulting in lower myelin thickness and shorter internodes, which are the portions of nerve fiber between two nodes of Ranvier [16]. These modifications might explain CMT1A’s reduced conduction velocity. Secondly, duplication of *PMP22* also appears to boost the expression of P2RX7, a purinergic receptor, which raises calcium levels in mature Schwann cells. In immature Schwann cells, calcium levels are high, and they are reduced as the Schwann cells develop. The myelin formation begins when the calcium levels are reduced, and the Schwann cells mature. When *PMP22* is overexpressed, P2RX7 receptor boosts the increase in calcium levels with the subsequent accumulation of calcium resulting in segmental demyelination [13]. Furthermore, deregulation of MAG and Necl4 proteins, which are important in sustaining axonal integrity, has been revealed [32], while it is found that the disruption of the Schwann-glia connection may result in axonal degeneration [20].

The symptoms of CMT1 can vary from mild to severe, although some patients are asymptomatic [18]. This range of phenotypic traits probably depends on the type of mutation. The destroyed myelin sheath, due to the absence of functional PMP22, affects both motor and sensory nerves, so the symptoms include difficulties in movements and senses. In most cases, symmetrical progressive muscle weakness and atrophy are present, eventually leading to limb deformities [14]. Considering the sensibility, loss of sensation is common among CMT1 patients, which usually includes no sense of vibration, pain or temperature. Additionally, deep tendon reflexes, particularly the Achilles and patellar reflexes, are frequently diminished or absent, due to nerve disfunction and muscle atrophy [33]. These symptoms usually appear in puberty or early adulthood, but in some patients, there is no suspicion of the disease until later in life [18]. Due to the complexity and the diversity of the disease, CMT1 is still incurable, but the genetic understanding accompanied by the pharmacological improvements promises life improvement and even therapy in the future [19].

#### 2.1.3. Hereditary Polyneuropathy with Liability in Pressure Palsies (HNPP)

HNPP is another polyneuropathy caused by mutations in *PMP22* and it is found to be inherited as an autosomal dominant trait [34]. Actually, HNPP and CMT1A serve as examples of genomic disorders, where certain structural features of chromosomes make them more prone to DNA rearrangements that can lead to disease [21]. Another remarkable correlation with CMT1 is the point mutation p. Thr118Met, which is found to cause HNPP in heterozygous patients [19], but the same mutation causes CMT1A in homozygous patients [18]. It is only in HNPP phenotype that a dominant G to A point mutation at position 202 in exon 3 leads to a Val30Met substitution [20].

HNPP is genetically similar to CMT1, as the same locus with the aforementioned polyneuropathy, located in chromosome 17, 17p11.2–p12, is in this case deleted [19]. In contrast to CMT1, in which overexpression causes a gain-of function phenotype, this polyneuropathy leads to a loss-of function phenotype, underlining the normal physiological functions of PMP22 [11]. The decreased amount of PMP22 that is produced in this pathologic state is insufficient for myelin formation and therefore, the axons are vulnerable to mechanical damage. This reduction in PMP22 disrupts the organization of tight junctions and adherens junctions between Schwann cells, increasing the permeability of the myelin sheath and making peripheral nerves more susceptible to pressure-induced injury [35].

Although most HNPP cases have the same genetic flaw, clearly, there are variations in clinical presentation. Many carriers are asymptomatic, but they can be traced by electrophysiological examination [36]. Reduced PMP22 protein levels and its association with other myelin components could result in varying degrees of severity in individuals, including asymptomatic gene carriers, HNPP patients, and those with chronic peripheral neuropathy [31]. The mild form of this disorder could possibly be attributed to the presence of PMP22. Most HNPP patients appear to have recurrent muscle weakness and sensory loss in particular nerves or plexuses with the peroneal and the ulnar nerves being primarily affected [37]. As a natural consequence of the pressure and compression, the nerves are subjected to gradual weakening over time. Limping and refusal to walk may appear early in life, but in some cases these symptoms appear only after the fourth decade [38]. Recurrent and self- limited episodes of peripheral nerve paralysis are present, usually following mild trauma at typical sites of compression [39]. Nerve biopsies exhibit distinctive localized thickening of the myelin sheaths (tomacula) [35], signs of segmental de- and re-myelination and a variable axonal loss owing to degeneration [36]. Diagnostic criteria include a significant slowing of motor and sensory conduction velocity in clinically affected, but also in clinically unaffected nerves [34]. Progressive sensorineural hearing impairment (SNHI) may also occur postanal at about age of 11 years old [40]. Until now, there is no complete therapy and only some preventative measures are used, such as AFO (Ankle Foot Orthoses), a wearable device which helps in gait and generally in the function of the lower limb, in order to improve the quality of life. Recent studies pointed out steroids, and particularly solumedrol, as possible treatment. In fact, patients who received them had a full recovery [41].

#### 2.1.4. Dejerine-Sottas Syndrome (DSS) and Congenital Hypomyelination Neuropathy (CHN)

As with most polyneuropathies, DSS is a sensory and motor neuropathy, quite complicated at a molecular and clinical level. A Ser to Leu substitution, resulting from a point mutation at nucleotide 264 in exon 3 of *PMP22* is known to cause this condition [22]. Same phenotype may also result from a His to Gln substitution at code 12, which comes from a C to A transition at nucleotide 85 [42]. Apart from multiple mutations in exon 3 of *PMP22,* which are inherited as an autosomal recessive trait, dominant point mutations are also present [27]. Several examples, such as Met69Lys, Ser72Leu, Leu16Pro and Leu80Arg are identified in heterozygous DSS patients, which proves that DSS can be inherited as a dominant trait [25,27], with the Ser72Leu being one of the most prominent variations [26]. Concerning CHN, a rare subcategory of DSS, molecular analysis has shown a T to C transition at nucleotide 374 in exon 3, leading to an Arg109Cys substitution, in the third transmembrane domain. The patient was heterozygous for this trait, indicating that the mutation is inherited in an autosomal dominant manner [28].

As a consequence of mutations, a supernumerary of Schwann cells is noted, causing onion bulb formation and thickening of the myelin sheath, in addition to demyelination and remyelination, which are observable in histological samples [23].CHN nerve biopsies have shown hypomyelination and basal laminae onion bulbs in all fibers. There is no progressive evolution noted, and so the disorder is characterised by myelin formation deficiency, rather than myelin instability [29]. The molecular and cellular changes caused by these mutations directly affect the structure and function of peripheral nerves, highlighting the complex pathophysiology underlying both DSS and its severe variant, CHN [28].

Although the symptoms of DSS are similar to CMT1, in this case the disease exhibits an earlier onset and with greater morbidity [43]. Affected individuals with DSS report severe weakness and atrophy of upper and lower limbs, hypesthesia, areflexia and thoracolumbar kyphoscoliosis [22]. Quite often, the severeness of the disease eventually leaves no option but the use of a wheelchair [25]. Patients with the Ser72Leu mutation exhibit also mild ptosis and limitations of eye movements [26]. CHN presents earlier compared to DSS and is recognised by slow nerve conduction velocity (NCV) and areflexia, while distal muscle weakness is also present [44]. Patients may exhibit the severe form, associated with joint contractures [45], or a milder form, with symptoms similar to DSS, making distinction between the two neuropathies difficult. Consequently, diagnosis relies mainly on sural nerve biopsies, as pathological and electrophysiological findings are often inadequate [46].

#### 2.1.5. Roussy–Levy Syndrome (RLS)

RLS is a rare HPN, considered as a subtype of CMT1B due to the hypertrophic and demyelinating lesions found during the nerve biopsy [47]. It is noticeable that 17p11.2 duplication of *PMP22* may also cause RLS, in addition to CMT1. Clinical research on 61 patients with a 17p11.2 duplication revealed that 50 of them have the CMT1 phenotype, whereas only eight suffer from RLS [14]. The duplication has usually paternal origin and less frequently maternal [48]. Like CMT1, RLS is inherited as an autosomal dominant trait [49]. Interestingly, this duplication appears to be a common locus for other disorders, with the same region in chromosome 17 implicated in Potocki-Lupski syndrome (PLS) and Smith-Magenis syndrome (SMS), both associated with neurological symptoms but differing from the typical polyneuropathy phenotype [50].

The pathophysiology of RLS mirrors that of other *PMP22*-related neuropathies. Like CMT1, RLS is characterized by slow nerve conduction, demyelination of nerve fibers, and the presence of onion bulbs in nerve biopsy specimens [51]. The hypertrophic and demyelinating changes seen in RLS mirror those observed in other *PMP22*-related neuropathies, linking the molecular alterations caused by the 17p11.2 duplication to structural abnormalities in peripheral nerves. These changes explain the electrophysiological features and morphological hallmarks of the disorder, highlighting the shared pathogenic mechanisms between RLS and CMT1 despite differences in clinical expression.

Clinically, RLS presents as a hereditary ataxia with features overlapping CMT1, with tremor as the predominant symptom [47]. Unlike most CMTs, disease progression is minimal or absent over time [52]. Muscular atrophy, weakness and loss of reflexion are present, with symptoms typically appearing in the first two years of life, and progression is slower [49]. This slow development is responsible for high life expectancy, with four out of five individuals living to be over 80 years old and remaining able to walk independently by the age of 70. Patients also develop bilateral pes cavus, wide-based and ataxic gait, and areflexia, all in early onset [51]. Mild scoliosis of the lower thoracic spine is another common symptom, while loss of vibratory and position sense in upper and lower limbs has been observed [52].

### 2.2. Myelin Protein Zero Gene (MPZ)

#### 2.2.1. Overview

Myelin protein zero (MPZ) is an integral membrane glycoprotein, which also plays a crucial role for the formation of the myelin sheath, consisting over 50% of the myelin substance [53]. Encoded by the *MPZ* gene, the protein is involved in the synthesis and the following compaction of the myelin sheath [54].The normally produced MPZ proteins attach to one other by homophilic interactions on Schwann cell membranes, allowing the arrangement of several layers of myelin lamellae [55]. Additionally, MPZ participates in signal transduction pathways, in order to regulate myelin formation and maintenance [55]. Apart from that, this molecule also interacts with other myelin proteins, such as PMP22 and myelin basic protein (MBP), ultimately contributing to myelin formation [56]. It has been proved that the specificity and inducibility are controlled by the c-acting elements of the gene, like the transcriptional factor Sox10 [57]. Multiple mutations of *MPZ* may result in the production of an abnormal protein, whose loss of function is threatening for PNS, causing numerous HPNs. It is noticeable that mutations that interfere with MPZ structure produce early-onset neuropathy, whereas those that affect MPZ-mediated transmission of signal and Schwann cell axonal interactions cause late-onset neuropathy [58].

Schwann cells only express *MPZ* when they are in close contact with the appropriate peripheral axons [59]. This interaction has two functional consequences, both of which enhance the myelin formation and stabilization: an increase in Schwann cells plasma membrane biosynthesis and the induction of other proteins, unique to myelin–forming cells, like myelin basic protein (MBP), myelin-associated glycoprotein (MAG), connexin 32 (CX32), and e-cadherin [54]. Once myelination is complete, its preservation is dependent on ongoing Schwann cell–axonal interactions. If a peripheral nerve is severed, separating the axon and its Schwann cells from the neuronal cell body, the axons degenerate and demyelinate, triggering an active process of anterograde degeneration, called Wallerian degeneration. This process concerns the distal end of the affected axon, as a consequence of a nerve lesion. After nerve injury, macrophages are among the first (2–3 days post-injury) and the most abundant cells to infiltrate the injury site, where monocytes/macrophages are recruited by factors produced by repair Schwann cells, and they then further produce chemoattractants, such as CCL2, TNF-α, IL-1α, and IL-1β, for greater macrophage infiltration. Macrophages appear to closely interact with the vascular compartments in order to drive the inflammatory process, as they selectively sense local hypoxia and secrete VEGF to polarize the neighboring vasculature [60]. During Wallerian degeneration, myelinating Schwann cells alter their gene expression pattern, turning off *MPZ* and other myelin-specific genes while activating a series of genes, includinglow-affinity nerve growth factor receptor (*NGFR*), previously expressed in immature Schwann cells prior to myelination [61]. CMT1B, DSS, and RLS are the most common phenotypes of *MPZ* mutation, as described below. Genetic alterations in *MPZ* that cause PNPs are listed in Table 2.

#### 2.2.2. Charcot-Marie-Tooth Disease Type 1B (CMT1B)

CMT1B is the second most common form of the group of autosomal dominant hereditary demyelinating neuropathies [71]. Linkage analysis, a gene-hunting technique that uses genetic markers that attach to known chromosomal locations, was applied to chromosome 1q21-23 and showed that this locus is associated with CMT1B [30,46].

Minor mutations in exons 2 and 3 of *MPZ* lead to a single amino acid change, i.e., a substitution or deletion in the extracellular domain of the protein [30]. More specifically, research on patients has shown a C to T transition at position 277 in exon 2, while in another case was found a G to A transition at position 444 in exon 3 [30]. In the same study, sequence analysis of exon 4 detected a single base change at positions 587 and 506, which led to the replacement of the codons Tyrl81 and Tyr154 by a stop codon, resulting in an aberrant MPZ protein. CMT1B may also arise from an Arg98Cys transition and a Ser63 deletion [64]. Studies on mice have reported Ser63del and Arg98Cys in vivo [72].

Although the genetic mechanisms behind CMT1B are not fully understood, most mutations in the *MPZ* gene result in neuropathy by a gain-of-function mutation [68]. This can happen through a variety of processes, including endoplasmic reticulum (ER) retention, activation of the unfolded protein response (UPR), mis-glycosylation and disruption of myelin compaction, and mistrafficking of mutant MPZ to the myelin sheath [62]. Notably, activation of the UPR due to misfolded proteins, leads to demyelination, a mechanism that equally applies to PMP22-related activation of pathological mechanisms. The mutant MPZ remains in the ER rather than being transported to the cell membrane or myelin sheath. Only a few mutations are connected with a loss of function pathway in this illness, resulting in milder neuropathy symptoms [73]. Symptoms in CMT1B can occur early or late onset, depending on the mutation. Mutations connected to an early onset lead to amino acid transitions such as Thr5Ile, Ser34Phe, Asp61Glu, Arg69Ser and Arg69Cys, whereas other transitions such as Asp6Tyr, Ser22Phe, Phe35del, and Glu42stop are connected with a late-onset phenotype [58].

CMT1B phenotypes vary in severity and can appear early in infancy or childhood with symptoms such as muscular weakness, foot abnormalities, sensory loss, and discomfort [74]. This condition can also emerge late in adulthood with the same symptoms [75]. Patients with CMT1B usually exhibit one of three distinct phenotypes: one with extremely slow NCVs and onset of symptoms during the period of motor development; a second with normal or near normal NCVs and the onset of symptoms as adults; and a third with normal development, with symptoms beginning gradually within the first two decades of life and slow NCVs similar to those found with CMT1A. In addition to the three classic phenotypes, individual symptoms are described. Clinical examination of a young patient revealed distal muscle atrophy and bilateral weakness, absent tendon reflexes, pes cavus, and decreased sensation in distal extremities [71]. This patient had a missense G to A mutation in exon 3, leading to the replacement of amino acid Arg by His at codon position 98 of MPZ protein. An older patient in the same study exhibited bilateral facial weakness and absence of vibratory sensation in both legs; he was unable to walk independently later in life, in addition to all the other symptoms that were similar between the two patients. Sequencing analysis of a third patient of the same study revealed a G to A transition in exon 3, leading to the replacement of Asp by Asn at codon 109, without altering a restriction enzyme site [76].

#### 2.2.3. Dejerine–Sottas Syndrome (DSS) and Congenital Hypomyelinating Neuropathy (CHN)

DSS is an infrequent yet significant disease, arising from mutations in *MPZ*. It is estimated that approximately 5% of *MPZ* mutations in the general population are accountable for this phenotype, specifically type 1B [63]. Several substitutions in the *MPZ* gene, such as Lys130Arg, Thr34Ile, Ser54Cys, Ile135Leu, Arg98Cys, and Ile62Phe, result in this phenotype, all following the autosomal dominant type of inheritance [25,65]. Another known substitution is Cys63Ser, and the literature also mentions a Arg168Glyc substitution [66]. Investigation of patients revealed a missense mutation resulting in a Arg69Cys amino acid change in the extracellular domain of MPZ and two frameshift mutations, one causing amino acid change after Leu-145 in the transmembrane domain of the protein, and the other after Gly-74 in the extracellular domain [46]. Several amino acid changes, such as Ile1Met, Ile33Phe, Ser34Cys, Tyr53Cys, Lys101Arg, and Ile106Thr, are responsible for the appearance of early-onset DSS with a mild to severe phenotype [58]. Interestingly, a three base-pair deletion in exon 2 that results in the deletion of Phe64 in *MPZ* causes DSS in homozygosity, but CMT in heterozygosity [67].

Mutations in *MPZ* cause DSS by disrupting both the structural integrity and intracellular processing of MPZ in Schwann cells. Those missense mutations that change the extracellular domain’s amino acid composition in ways that impair the homophilic adhesion between MPZ molecules, leading to defective myelin compaction [68]. In contrast, mutations that especially that produce premature stop codons, escape nonsense-mediated mRNA decay and yield truncated proteins that act in a dominant negative way, interfering with normal MPZ function [69]. Additionally, accumulation of misfolded MPZ in the endoplasmic reticulum triggers the UPR, leading to Schwann cell stress and further compromising myelination, like in CMT1B [63].

In addition to DSS patients, heterozygous patients with CHN who have certain mutations in *MPZ* have been identified. One amongst them is a nonsense mutation of the gene affecting the intracellular domain of the protein in position 186, replacing Gln amino acid and creating a termination codon [46].

#### 2.2.4. Roussy–Levy Syndrome (RLS)

A few mutations in *MPZ* are accountable for the RLS phenotype. A heterozygous missense C to A transition in position 727 in exon 3, leading to an Arg131Lys substitution, is the most frequent cause of this neuropathy [52]. It is also found that a C to T point mutation at position 428, leads to the Thr143Met substitution in the protein. This mutation affects the myelin structure, contributing to the motor and sensory neuropathies seen in RLS [51]. Moreover, a Asn102Lys transition is responsible for delayed development of RLS, at about 5 years old, but with severe symptoms appearing later in life [58].

### 2.3. Mitofusin 2 Gene (MFN2)

#### 2.3.1. Overview

*MFN2* is a protein-coding gene directly involved in the onset of CMT2A. A study of seven families affected by CMT2A found that all had missense mutations in this gene [77]. MFN2 exhibits dimerization-stimulated GTPase activity, involving multiple cycles of GTP binding, detachment, and hydrolysis. The protein has a basal (low) GTP turnover, a natural GTP hydrolysis, which is crucial for its cellular function, as several GTPase-promoting mutations are linked to CMT2A onset [78]. There are various mechanisms behind *MFN2*-related CMT2A. Some dimerization-deficient mutations, while promoting mitochondrial fusion, have minimal impact on wild-type *MFN2*. These mutations result in unfolded or truncated proteins that cannot form dimers. In contrast, other mutants can still dimerize and retain some fusogenic activity, but they often have a dominant negative effect on MFN2, disrupting its weakened activity. The level of retained fusogenic activity in these mutants plays a crucial role in disease onset [79]. Furthermore, several mutations in MFN2’s GTPase domain, including Arg94Gln, Arg104Trp, Thr236Met, Ser249Cys, and Arg280His, reduce the ability to hydrolyze GTP to GDP, the last step in mitochondrial fusion. As a result, shorter and depolarized “clumped mitochondria” are formed, which stay linked together but are unable to perform fusion due to the absence of GTPase activity [78].

Its protein product, called MFN2, is a dynamin-like GTPase located on the outer mitochondrial membrane, where it plays a key role in maintaining mitochondrial structure by facilitating the fusion of mitochondrial membranes, which is essential for proper mitochondrial network organization [33]. This GTP-dependent process merges the outer and inner mitochondrial membranes, facilitating the exchange of substances like oxygen, which indirectly shapes and structures the mitochondrial architecture [80]. MFN2 deficiency reduces fuel oxidation and the mitochondrial membrane potential, leading to oxidative stress, impaired metabolism, and eventually, cell death, due to disruption of the proton gradient and intramembrane voltage necessary for ATP synthesis [81]. In loss-of-function experiments with mice, Mfn2 deficiency decreased Krebs cycle and electron transport chain activity, leading to increased glucose uptake and glycolysis but reduced glycogen synthesis [82]. These metabolic disruptions are especially harmful in highly oxidative tissues like the nervous system, skeletal muscle, and heart, triggering mechanisms linked to disease pathogenesis [83]. An Arg400Gln substitution has been shown to cause perinatal cardiomyopathy, characterized by abnormal mitochondrial morphology, disrupted mitophagy, and impaired mitochondrial quality control. These findings suggest that, in addition to its well-established role in peripheral neuropathy, MFN2 dysfunction may contribute to cardiac disease through defects in mitochondrial maintenance and energy metabolism [84]. *MFN2* is crucial for neuron and Schwann cell function, along with other genes related to polyneuropathies.

#### 2.3.2. Charcot–Marie–Tooth Disease Type 2 (CMT2)

CMT2 is a hereditary axonal neuropathy that causes the death of axons of peripheral nerves. *MFN2* has been reported as the most commonly associated gene in this form of CMT, presenting with an autosomal dominant type of inheritance [85]. Several mutations in *MFN2* have been detected, with the C to T transition at position 280 in exon 4 being the most common among CMT2 mice samples and also recorded in patients [86]. A C to G nucleotide change at position 299 in exon 4 leads to another substitution, A100G, responsible for the disease [87]. A number of loss-of-function mutations of the same gene affect the GTP-associated domain of MFN2. For instance, a G to A change at position 281 results in an Arg to Gly amino acid substitution, while a G to C amino acid change at position 2219 leads to a substitution of the aromatic Trp to the small polar Ser [77]. GTP-domain mutations also include a C to T change at nucleotide 748, causing an Arg250Trp substitution. Similarly, a C to T change in position 368 of exon 5 causes a P123L amino acid change and an A to G transition at 494 positions in exon 6 leads to a H165R substitution. In addition, two C to T nucleotide changes, one at position 493 and the other at position 617 are recorded, resulting in H165Y and T206I amino acid changes, respectively [87]. A G to A transition in position 839 in exon 9 is also reported, leading to a Arg280His substitution. In the same study, a C to T change at nucleotide 1081 has been detected in participants, while others have a G to A change at site 1128. It seems that the C to T transition is a common point mutation for the CMT2 phenotype, as two more have been reported, one in position 1198 of exon 12, changing the Arg at position 400 into a stop codon, and the other in position 2251 of exon 19, which leads to a nonsense mutation. Apart from single-nucleotide mutations, there are cases of dinucleotide changes, such as an A to C and a G to T change in positions 1157 and 1158 consecutively, occurring in cis amino acids, with Gln residue at position 386 being replaced by Pro [87,88]. It is also known that a Thr105Met and a Pro251Leu amino acid changes results in a lack of fusogenic activity, which prompts the neuropathy [79].

Structurally, MFN2 consists of an amino-terminal GTPase domain, a first coiled-coil heptad repeat region (HR1), two transmembrane domains, and a carboxyl-terminal heptad repeat region (HR2). Normally, MFN2 alternates between a folded, inactive conformation where HR2 binds intramolecularly to HR1, and an unfolded, active conformation in which HR2 extends into the cytosol to promote mitochondrial tethering and fusion [89]. This conformational switch is critically regulated by amino acids within the HR1 domain, particularly, Met376, Ser378, His380, and Met381, which stabilize or destabilize the HR1–HR2 interaction. Mutations that alter these residues or nearby regions can trap MFN2 in a closed, fusion-incompetent state, leading to mitochondrial fragmentation [90]. Disease-associated mutations such as a T to M change at position 105 or a K to A change at position 109 further disrupt this conformational flexibility or the associated GTPase activity, preventing HR2 from extending and thereby impairing mitochondrial fusion [89]. Moreover, mutations in the GTPase domain impair GTP hydrolysis, allowing mitochondria to tether but preventing fusion, resulting in short, depolarized, and perinuclear-clumped mitochondria. In contrast, HR1 domain mutations disrupt MFN2 conformational changes while preserving GTPase activity, producing small mitochondria without depolarization or perinuclear aggregation [91]. These findings highlight distinct domain-specific mechanisms. GTPase mutations block the final fusion step, whereas HR1 mutations prevent mitochondrial tethering, leading to the structural instability characteristic of CMT2A pathology [89].

Nerve biopsy findings of this disease reveal chronic axonal atrophy and regeneration with very slow progression, while slightly reduced motor and sensory NCVs are present, accompanied by decreased amplitudes of evoked motor and sensory nerve responses [92]. According to these findings, loss of MFN2 function causes axonopathy possibly by impairing energy production along the axon [82]. Appearing in early childhood, sural nerve biopsy shows variable loss of myelinated fibers, mainly of large diameter, while small onion bulb-like structures are often observed [93].

**Figure 1:**
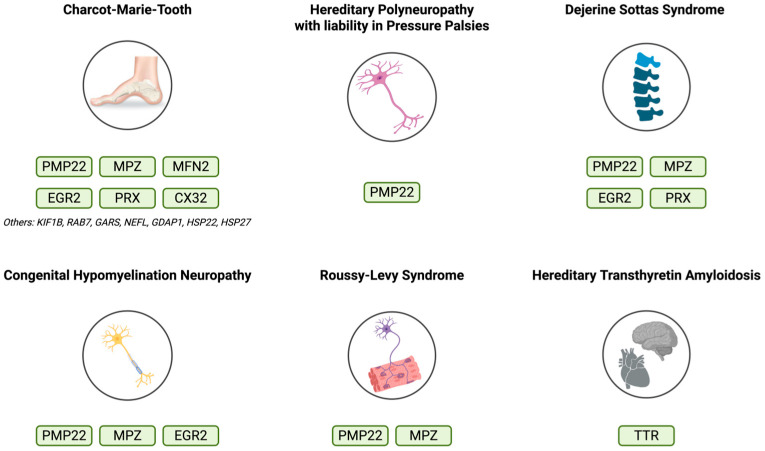
Principal genes associated with hereditary polyneuropathies. PMP22: Peripheral myelin protein 22. MPZ: Myelin protein zero, MFN2: Mitofusin 2, EGR2: Early growth response protein 2, PRX: Periaxin, Cx32: Connexin 32, TTR: Transthyretin. Created in BioRender. Dermitzakis, I. (2026) https://BioRender.com/5fedbas.

Compared to CMT1, patients with CMT2 exhibit a more severe clinical picture with greater severity of disability [94]. As the main symptom, CMT2 patients present difficulty in walking, due to deformities of the lower limbs. Tendon areflexia is present in most cases, as well as muscle weakness, but sensory loss of vibration sense is, also, detected in about half the cases of CMT2 [95]. Patients also report leg muscle cramps and pes cavus, meaning a foot with an abnormally high plantar longitudinal arch, while frequent falls may occur due to the lack of muscle strength, which eventually progresses to total distal paralysis [87]. It is remarkable that in many cases the upper limb seems unaffected, whereas in other patient’s finger tremor and sensory loss of all senses have been reported [96]. The key genes responsible for HNPs are summarized in Figure 1.

### 2.4. Transthyretin (TTR)

#### 2.4.1. Overview

*TTR* is a protein coding gene, whose product is called transthyretin (TTR). TTR is found in plasma, when secreted by the liver, and cerebral fluid, when secreted by the choroid plexus, and delivers thyroxine (T4) and retinol to the liver. Research conducted in knockout (KO) mice, in which *TTR* had been inactivated, has proved the role of TTR in maintaining normal levels of metabolites, such as serum retinol, RBP, and thyroid hormone in plasma [97]. The transport of thyroid hormones and vitamin A seems to be the conservative function of this protein, but not the only one. Recent research suggests that TTR may have a function in neuroprotection and neurite outgrowth after nerve damage, by increasing the levels of lipoprotein lipase and sphingolipids [98]. Absence of TTR was associated with sensorimotor impairment and delayed functional recovery following a sciatic nerve crush, demonstrating the role of TTR in peripheral nerve function and repair [99].

In addition to the peripheral nervous system, TTR deficiency significantly affects the central nervous system (CNS), as TTR modulation of the noradrenergic system entails behavioral alterations like reduced signs of depressive-like behavior and increased exploratory activity [100]. TTR has an additional protective role in the CNS; it is found to be increased after brain injury due to ischemia. An experimental method called permanent middle cerebral artery distal occlusion (pMCAO), demonstrated on CO mice, proved that TTR causes cerebral edema formation after focal cerebral ischemia in mice [101]. SiRNA experiments support TTR circulating in CSF and crossing the CSF/brain interface. CSF-TTR diffusing into the brain interstitium would then infiltrate the ischemic tissue entering injured neurons, committed to die [101].

Focusing on the TTR’s effect in memory, high levels of the protein are associated with good memory in young mice, while reduced TTR levels are detected in aged mice with memory gaps [102]. Moreover, KO mice present impaired memory, in contrast to mice that produce normal levels of this protein [103]. Research is not limited to animal model studies but has expanded to include clinical trials. This suggests that TTR may be an independent predictor of positive clinical outcome, estimating that high serum concentration of TTR may be a prognostic indicator for the outcome of cerebral infarction [104]. Interestingly, TTR has been suggested to protect against Alzheimer’s disease (AD); in AD, amyloid-b (αβ) plaques accumulate in the brain, causing gradual loss of neurologic function in AD. The early indication of a function for TTR in AD is derived from the fact that when αβ was introduced to the CSF of patients and controls, it was sequestered by TTR, which is the most abundant αβ-binding protein in the CSF [105]. Moreover, several reports have described decreased levels of TTR in the CSF of AD patients [106]. Levels of CSF TTR in AD patients have been shown to negatively correlate with the degree of dementia [107]. Recently, evaluation of TTR levels in human serum showed a negative correlation between serum TTR levels and AD, as human serum TTR levels were shown to be decreased in AD patients compared to non-demented controls [108].

#### 2.4.2. Hereditary Transthyretin Amyloidosis (hTTR)

*TTR* is associated with the appearance of hTTR, an autosomal dominant neuropathy [108]. Mutations in *TTR* are responsible for the appearance of hTTR, a progressive, debilitating disease that is ultimately fatal. It is characterized by a misfolding of TTR, predominantly affecting the small fibers. NCVs are slow and show axonal damage with prolonged distal motor latencies in all nerves tested [109,110]. More than 120 pathogenic *TTR* mutations have been reported worldwide, but the two most frequent are the Val30Met and the Phe64Leu trasitions in the *TTR* gene [109]. Other mutations that are listed as causes of hTTR include Val122Ile, Thr60Ala, and Ala120Ser [111]. The symptoms usually differ, depending on the type of mutation and may or may not include common neurological symptoms like other neuropathies. Patients with the Val30Met and Phe64Leu substitutions report lower limb paresthesia, while cardiomyopathy is also present [109]. This certain phenotype includes autonomic involvement, such as impotence, gastrointestinal symptoms, postural hypotension, and bladder dysfunction, all occurring usually on early onset, but the progression is slow [112]. Early-onset patients usually mention having paresthesia, burning and extending pain in the feet, dysesthesias, and dysautonomia including impotence, diarrhea, or constipation as symptoms. They also exhibit a progressive sensory–motor and autonomic neuropathy, with an expected survival of approximately 10–20 years from onset. By contrast, late-onset Val30Met patients, as well as non-Val30Met cases, have usually a more rapid and severe progression and are associated with a median survival of nearly 7 years since the disease onset [113]. Patients with other mutations such as Val122Ile, Thr60Ala and Ala120Ser, present a multisystem disease, as hTTR affects the heart, the kidney and the ocular vitreous, while symptoms related to peripheral neuropathy are present at about 54% of those cases [111].

### 2.5. Early Growth Response Protein 2 Gene (EGR2)

#### 2.5.1. Overview

*EGR2* belongs to the *EGR* family, which includes genes that encode proteins essential for cellular proliferation. Specifically, these genes encode Cys2-His2 type zinc-finger proteins, which are widespread DNA binding molecules eukaryotic transcription factors and play a crucial role in gene expression, cell development and differentiation [114]. *EGR2* belongs to c-fos-like genes, which activate rapidly as a response to a stimulus by mitogens, like growth factors. This mechanism is called fos-like kinetics [115]. Due to their structure and fos-like kinetics by diverse mitogens, EGR proteins act as third messengers by coupling early biochemical processes to long-term changes in gene expression required to modulate cell growth [116]. By influencing which genes are turned on or off, EGR proteins help the cell transition from an initial response to sustained physiological changes [117].

*EGR2* is expressed in several cell types, with fibroblasts and lymphocytes being the main ones. Research on mice suggested that *EGR2* is another gene associated with myelin formation in PNS, along with the other genes mentioned previously. It is indicated that *Egr2* is associated with the formation of myelination in the PNS, whereas Schwann-cell differentiation at an early stage is intercepted in homozygous knockout *egr2* mice [118]. The speculation that EGR2 acts as a transcription factor in response to various stimuli, including growth factors, stress, and mitogens, means that this protein affects myelin genes and hence, peripheral neuropathies may result from mutations in it [119]. Sequence analysis showed that *EGR2* causes autosomal dominant loss-of-function mutations in human patients, while microarray expression analysis indicated EGR2 as an essential transcription factor for myelin-protein-encoding genes like *PMP22*, *MPZ*, *PRX*, *CX32*, and *MBP* [120]. Most of these mutations are located within the DNA binding domain (DBD) and exhibit greatly decreased (or absent) DNA binding and transcriptional activity [121].

Mutations in *EGR2* are strongly implicated in HPNs, including CMT1, DSS, and CHN. The location of the mutation in *EGR2*, and the effect in protein function determine the type of peripheral neuropathies, as well as the severity of the disorder. *EGR2* was suggested to cause HPNs after being obtained in studies of mice containing mutations in this transcription factor. While mice heterozygous for *Egr2* were phenotypically normal, the ones deficient in this gene had severe demyelination of peripheral nerves [122]. These findings highlight the crucial role of *EGR2* in myelin gene regulation and underscore how its disruption leads to clinically significant demyelinating neuropathies.

Mechanistically, EGR2 functions as a transcription factor that together with SOX10, another transcription factor in Schwann cells, orchestrates the expression of key myelin genes such as *MPZ*, *MAG*, and *PMP22*, while also interacting with NAB co-repressors to fine-tune transcriptional activity [123,124]. Mutations in *EGR2* disrupt these interactions, leading to reduced transcriptional activation of myelin-related genes. Beyond transcriptional regulation, EGR2 also directs histone H2B monoubiquitination via the RNF20/RNF40 complex, an epigenetic modification essential for proper chromatin accessibility and myelin gene expression. When this epigenetic control is lost due to pathogenic variants, Schwann cells fail to fully differentiate, resulting in defective myelination [125].

Several mutations in the two exons of *EGR2* have been found to result in CMT1. Direct sequencing of the gene revealed a heterozygous A to T transversion at nucleotide 1064 that predicts an Asp305Val substitution within the first zinc-finger domain. This pathogenic mutation leads to a severe CMT1 phenotype [123]. Another heterozygous mutation is a C to T transition, predicting an Arg409Trp amino acid substitution within the third zinc-finger domain [121]. Patients with CMT1 clinical diagnosis who did not present certain mutations in *PMP22* or *MPZ* were the motive for further research on *EGR2*. It was found that the same phenotype may result from an Arg382Tyr substitution, affecting the a-helix of the second zinc finger domain of *EGR2* [126]. Several other mutations in *EGR2* can result in a CMT1 phenotype, such as Arg359Gln [127], Arg381Leu [126], Thr387Asn [128], and Glu412Gly [129]. Moreover, an Arg381His amino acid change is regarded as another cause for this sensory and motor neuropathy [130], while an Arg381Cys substitution occurring at the zinc-finger 2 domain is suggested as a possible cause of CMT1 [131]. Located in the first zinc-finger domain, the substitution Arg353Gly is also recorded in CMT1 patients [132].

#### 2.5.2. Dejerine- Sottas Syndrome (DSS) and Congenital Hypomyelination Neuropathy (CHN)

DSS may also arise from mutations in *EGR2*. A known cause is an A to G nucleotide change at position 1232, which leads to a missense p. Asp411Gly substitution, resulting eventually in a severe DSS phenotype without an early onset [133]. Moreover, a de novo C to T mutation at nucleotide 1075 is found to cause an Arg to Trp amino acid change at codon 359, affecting the a-helix of the first zink-finger domain of EGR2 [134]. A few more substitutions are found to cause DSS phenotype, such as Asp383Tyr [124] and Glu412Lys [129].

Moreover, mutations in the same gene are associated with a CHN phenotype. A sequencing analysis in an affected family showed a T to A transversion, leading to an Ile268Asn transition within an inhibitory domain. Concerning the pattern of inheritance, CHN is inherited as an autosomal recessive trait, as there are healthy family members who are heterozygous, while only members who are homozygous present symptoms. The above-mentioned mutation is speculated to provoke the dysregulation of *EGR2*, causing an increase in transcriptional activity [119]. Sequence analysis of cloned PCR products in another CHN patient has two different heterozygous mutations, determined to be on the same DNA strand. The first mutation is a T to G transversion, leading to Ser382Arg amino acid change, while the second one is a G to T nucleotide change that results in Asp383Tyr substitution [119]. Another cause of CHN seems to be an Ile268Asn amino acid change, occurring in the NAB repressor binding site domain of the EGR2. NAB refers to NGFI-A (Nerve Growth Factor-Induced A) binding proteins that are involved in regulating the transcriptional activities of EGR proteins by acting as co-repressors.

### 2.6. Periaxin Gene (PRX)

#### 2.6.1. Overview

Rare CMT cases, specifically type CMT4F, as well as DSS [135], may result from mutations in *PRX*, a coding gene whose product is associated with the stabilization of myelin in the PNS and particularly in Schwann cells [136]. Considering its role in cortical signaling, *Prx* encodes two proteins with PDZ domains, L-periaxin and S-periaxin. The PDZ motif is present in a variety of proteins, which are believed to have an organizing function at sites of cell-to-cell contact [137]. These proteins are implicated in the assembly of macromolecular signaling complexes, which contribute to the stabilization of Schwann cell cytoskeleton and the formation and maintenance of internodes, the regions between two nodes of Ranvier in myelinated axons [138]. Prx proteins may also interact with the cytoplasmic tail of transmembrane proteins, such as glutamate receptors. Certain mutations in the *PRX* gene lead to excessive myelin production, which is subsequently followed by cycles of demyelination and remyelination [139]. The precise mechanisms that regulate myelin sheath thickness remain unclear, but they are likely influenced by the axon, as the expression of the gene is controlled through axonal contact [140]. Studies on mice with a deficiency in functional *Prx* assemble compact PNS myelin. This results in an unstable myelin sheath, leading eventually to demyelination and reflex behaviors, accompanied by peripheral nerve damage and hence, pain [135,141]. These findings suggest that the periaxins are essential for the establishment of a stable Schwann-cell axon unit in the myelinated fibers of the PNS [141].

#### 2.6.2. Dejerine-Sottas Syndrome (DSS)

Most mutations of this gene that are associated with DSS polyneuropathy occur in exon 7 of *PRX* [142]. Two specific autosomal recessive inherited mutations were found in a Korean-population study; they both consist of a C to T transition, the first at nucleotide 1174 and the second at 2035. Both of them result in a codon that codes Arg transmuting to a stop codon [139]. Thus, it was postulated that both mutations lead to enzymatic defects, and that these compound heterozygous mutations were the underlying cause of the DSS phenotype. Regarding other mutations responsible for the disease, a homozygous mutation that leads to a frameshift mutation is known to cause a substitution of Arg at codon 96, affecting both S- and L-periaxin and causing DSS [135]. A couple of nonsense mutations in *PRX* have also been identified to cause DSS, resulting in an Arg changed by a stop codon, one at position 953 and the other at position 1070 [143]. The last one is derived from a C to T nucleotide change at position 3208 [144]. The defective L-periaxin and S-periaxin are unable to form complexes with other proteins and, consequently, both the cytoskeleton and myelin of Schwann cells are unstable, affecting their structure and the ability of rapid conduction of nerve signaling accordingly [145].

Concerning the clinical image, these patients confront hypotonia and distal muscle weakness from a very young age. The condition worsens gradually, with loss of sensation and areflexia being present. Foot deformities and scoliosis may also arise, eventually leading to the patient requiring walking aids or even a wheelchair [144]. *PRX*-associated neuropathies are characterized by slow clinical progression with the characteristic onion bulbs and axonal atrophy being present [143].

#### 2.6.3. Charcot-Marie-Tooth Disease Type 4F (CMT4F)

Certain mutations in *PRX* are identified as causes of CMT, and particularly the CMT4F subtype, which is an autosomal recessive, early-onset, demyelinating neuropathy [145]. One of the recorded mutations is a homozygous T to A mutation at nucleotide 2145 resulting in a Cys substituted by a stop codon at position 715. This affects the integrity of the L-periaxin and causes an early-onset, slowly progressing neuropathy [146]. A nonsense mutation resulting in an Arg to be substituted to a stop codon at position 196, has also been identified. Consequently, delay in development and distal muscle atrophy occur, accompanied by mild kyphoscoliosis, which are present in CMT4F phenotype. This condition is possibly derived from nonsense mutations, as there are two examples identified, a Cys715X and an Arg1070X relatively, while a frameshift mutation leads to an Ala700Pro substitution [145]. A homozygous C to T transition at position 860 in *PRX* leads to a premature stop codon at the beginning of the exon 7, in the place of an Arg. This nonsense mutation truncates the C-terminus of L-periaxin by 1266 amino acids and leads to the absence of a large repeat-rich domain and a C-terminal acidic domain, the functions of which are not yet fully characterized [145].

### 2.7. Connexin 32 (CX32 or GJB1)

#### 2.7.1. Overview

CX32 is a highly conserved protein that belongs to the family of connexins, and it is mainly produced in Schwann cells in the PNS, as well as in other tissues such as the CNS and the liver [147]. Known for its role in the PNS, CX32 ensures that Schwann cells are kept in close contact with the axons, creating contact-zones between them, where the protein is mostly active. In both nerves and ganglia of the PNS, CX32 localizes only in myelinating Schwann cells, mainly to the paranodes, the periodic interruptions in the compact myelin and the two outer layers of the myelin sheath [147]. *CX32* expression at these sites creates hemichannels that connect the cell’s cytoplasm with the extracellular medium. Specifically at these sites, CX32 creates reflexive gap connections across the myelin sheath, accelerating communication via the myelin layers that divide the adaxonal -or inner- and the abaxonal -or outer- layer of myelin sheath [148]. Furthermore, the ectopic release of ATP in Schwann cells is strongly dependent on CX32 [147], in an activity-dependent manner from the axons [149]. This means that the release of ATP in Schwann cells is not constant but instead depends on the activity level of the axons they surround [150]. When axons are more active, they signal Schwann cells to release ATP through CX32 channels. This activity-dependent ATP release can modulate the differentiation and proliferation of glial cells, including Schwann Cells [151]. Interestingly, it has been discovered that the expression of *cx32* in the PNS is regulated by the transcription factor EGR2 which directly binds to the *cx32* promoter with synergistic action [152].

#### 2.7.2. CMT1X-Linked Neuropathy

Several nonsense, missense, deletions and insertions in the *CX32* gene have been identified as mutations that possibly cause CMT1X neuropathy. Although all kinds of changes can occur in the same gene, the type of mutation determines the severity. Specifically, missense mutations result in a mild clinical phenotype, whereas nonsense mutations lead to severe phenotypes [95]. Certain mutations in *cx32* in mouse models are found to cause CMT, such as a nonsense C to T nucleotide change, which leads to an Arg changed by a stop codon, which eventually leads to a severe CMT clinical image. Several other mutations in this gene have also been identified. A G to A point mutation is followed by an Arg to Gln substitution at position 22, while a C to G transition results in a Trp to Ser transition at position 77. Moreover, two A to G transitions are identified in patients, the first leading to a Glu to Gly amino acid change at position 102 and the second leading to a Gln to Arg substitution at position 80. A C to T transition is also known for leading to an Arg to Trp amino acid change at position 142 [153], in contrast to a T to C transition that causes a Trp to Arg change at position 3, while a G to C change at the same position leads to a Trp to Ser change. Furthermore, a G to A point mutation contributes to an Arg to Gln change at position 22 and a C to T mutation results in Arg164Trp [154].

In addition to frameshift mutations, which usually cause mild symptoms, CMT1X-linked neuropathy may also be derived from nonsense mutations with more severity. A G to T transition results in Glu to a stop codon at position 186, while a T to A transition results in Cys to a stop codon at position 217 and a C to T transition leads to an Arg substituted by a stop codon at amino acid 220 [155]. Besides that, two deletions have been observed in patients with this neuropathy, leading to a moderate and a severe phenotype, respectively; the first encompasses 18 bp of DNA and includes 111–116 amino acids, while the second is larger, affecting 29 bp and amino acids 265–273 [95]. Loss of *CX32* dysregulates several genes associated with immune response, thus contributing to the severity of the disease [156]. Concerning CMT1X-linked neuropathy, mutations in this gene obstruct the formation of gap junctions that normally contribute to the transportation of ions and signal transmission between Schwann cells, leading to demyelination. Gap junctions normally play a role in stability of myelin, as well, and so, mutant connexins fail in the maintenance of myelin integrity and axonal damage. Gradually, this leads to progressive degeneration of nerve fibers [152].

This degeneration is accompanied by characteristic clinical symptoms of CMT1X, such as muscle weakness, sensory loss and atrophy. In contrast to all the other autosomal inherited polyneuropathies that are previously described, this form is X-linked, which means that affected males experience more pronounced weakness than affected females [156]. The type and location of *CX32* mutations determine the severity of the phenotype, ranging from mild to severe manifestations [95].

### 2.8. Other Genes Associated with HPNs

There is information about more than 80 other genes that are involved in some stages of myelin sheath formation of Schwann cells in PNS and therefore, are associated with certain forms of polyneuropathy, or other, yet undescribed neuropathies of PNS [157]. Five new genes and 13 predicted genes have been identified, most of which are expressed only in embryonic stages. Among them, research has focused on *tektin 4* (*TEKT4)*, *heparan sulfate-glucosamine 3-sulfotransferase 3B1* (*HS3ST3B1)* and *CMT1A duplicated region transcript 15* (*CDRT15)* [21]. Others revealed *Dynactin subunit 1 (DCTN1)* and *Rho guanine nucleotide exchange factor 10* (*ARHGEF10)*, as possible CMT-related genes [158].

Regarding CMT2, multiple genes are found to be associated with the several subtypes of CMT2. To begin with one already mentioned gene, certain mutations in *MPZ*, like Ser15Phe, Asp32Gly, Asp46Val, Tyr90Cys and Thr95Met, may result in mild CMT2 phenotype [71]. Besides that, an autosomal dominant mutation in *kinesin family member 1B* (*KIF1B)*, a protein coding gene located in chromosome 1 at p36.2 locus, is known for producing a mutant Kinesin family member 1B, blocking axonal transport, resulting in axonal degeneration [159]. Moreover, *Ras-related protein Rab-7a* (*RAB7)* in chromosome 3 encodes a Ras-associated protein, RAB7, which is implicated in endosomal trafficking. Mutations in this gene are described as autosomal dominant and are responsible for CMT2B appearance [160]. CMT2D phenotype may also result from mutations in *glycyl-tRNA synthetase* gene (*GARS)*, a gene located in chromosome 7 that produces a Glycyl-tRNA synthetase, essential for RNA processing [161]. Additionally, CMT2E is often caused by mutations in *neurofilament light chain (NEFL)*, a gene located in chromosome 8 that encodes NEFL, which plays a role in the neuronal cytoskeleton.

*Ganglioside-induced differentiation protein 1 gene (GDAP1)* is also located in chromosome 8, which encodes a ganglioside induced differentiation protein 1, known for its role as a mitochondrial glutathione transferase. Appearing autosomal recessive inheritance, mutations in this gene cause CMT4A phenotype, a quite common form of CMT [162]. There are some mutations recorded in *GDAP1* that are inherited as autosomal dominant variants and cause amino acid changes such as a Trp to Arg substitution at position 120. Patients with those mutations generally experience milder symptoms than the ones that carry autosomal recessive forms of this gene [163]. CMT2F subtype arises due to mutations in *heat shock protein family B member 1 gene (HSP27)*, which normally produces a small heat shock protein 27, known for its role in mitochondrial molecular chaperone and neurofilament assembly [164]. Similarly, CMT2L is a subtype of the disorder resulting from *heat shock protein family B member 8* gene (*HSP22)* mutations, a gene which normally encodes a small heat shock protein 22 [165], very similar to HSP27. Both of those proteins are found to protect against protein misfolding and prevent H2O2-mediated cell death [166]. Some of the earlier mentioned conditions are inherited exclusively as dominant traits, while others can appear as both dominant and recessive variants, which differentiates the clinical image [96]. A complete overview of the phenotype, clinical correlations, genes and molecular mechanisms associated with each HPN is presented in Table 3.

## 3. Gene-Linked Biological Pathways in the Pathogenesis of Hereditary Polyneuropathies

Some genes associated with HPNs share a similar pathological pathway, which renders the molecular characterization not only intriguing, but also more complicated. Among them, *PMP22* is the most prominent, as it is a dosage-sensitive gene whose copy number variants cause distinct forms of HPNs, like CMT1A and HNPP. These phenotypes result from duplication or deletion, respectively, of the 17p11.2–p12 locus [6,13]. When this locus is duplicated, generating *PMP22* overexpression, the regulation of myelin-related proteins becomes disrupted, ultimately suppressing key enzymes involved in cholesterol biosynthesis [17]. Such alterations profoundly affect myelination, during peripheral nerves development, resulting in reduced myelin thickness, shorter internodes and reduced NCV [16]. A further consequence of *PMP22* duplication appears to be the activation of P2RX7, which elevates intracellular calcium levels in Schwann cells and drives segmental demyelination [13]. Moreover, deregulation of MAG and Necl4 proteins along with perturbation of Schwann cell–axon interactions, compromises axonal integrity and promotes degeneration. Notably, duplication of 17p11.2–p12 region can also cause RLS, apart from CMT1. Although the clinical manifestations of these two conditions differ to some extent, they share the same underlying pathogenic mechanisms [14]. The question of why the majority of individuals with this duplication develop CMT1A, whereas only a small subset present with RLS, remains unresolved and warrants further research. Conversely, deletion of the same locus disrupts normal PMP22 function, resulting in a decreased protein product [11]. In HNPP pathology, the diminished production of PMP22 is insufficient for proper myelin formation, exposing the axons to mechanical stress. The lowered PMP22 levels impair the structure of tight and adherent junctions between Schwann cells, which increases myelin sheath permeability and heightens the vulnerability of peripheral nerves to pressure-related damage [35]. On the other hand, a wide range of point mutations in *PMP22* can cause either DSS, or its more severe variant, CHN. These mutations result in a supernumerary of Schwann cells that causes repeated demyelination–remyelination cycles, as well as abnormal thickening of the myelin sheath [23]. The resulting myelin formation deficiency, rather than myelin instability, directly impairs the structural and functional integrity of peripheral nerves, underscoring a complex pathophysiological process that needs further investigation [28].

All of the aforementioned disorders can also arise from *MPZ* gene mutations. CMT1B results from a molecular mechanism that is not yet fully elucidated, but current evidence implicates abnormal glycosylation, impaired myelin compaction, and retention of MPZ within the endoplasmic reticulum rather than proper trafficking to the cell membrane [62]. Notably, the accumulation of misfolded MPZ activates the UPR, leading to demyelination—a process similarly observed in PMP22-related pathology. Furthermore, certain *MPZ* mutations produce DSS in the homozygous state, whereas the same variants cause CMT in heterozygotes [67]. In DSS, the structural stability and intracellular handling of MPZ in Schwann cells are compromised, altering the amino acid composition of its extracellular domain and thereby disrupting the homophilic adhesion essential for its normal function. Conversely, mutations introducing premature stop codons evade nonsense-mediated decay and generate truncated MPZ proteins with dominant negative effects, interfering with normal MPZ function [69]. Despite involving distinct molecular routes, both mechanisms ultimately result in peripheral nerve demyelination. The UPR is activated in these contexts as well, inducing Schwann cell stress and further undermining effective myelination [76]. CMT2A is most associated with *MFN2* gene mutations. Alterations in the HR1 domain, particularly in regions that regulate its interaction with the HR2 domain, lock MFN2 into a closed, fusion-deficient conformation, resulting in pronounced mitochondrial fragmentation [91]. In this state, the HR2 domain cannot properly extend, thereby obstructing the mitochondrial fusion process [89]. Mutations affecting the GTPase domain impair GTP hydrolysis, allowing mitochondria to tether but preventing their fusion. As a consequence, mitochondria become shortened, lose membrane potential, and accumulate abnormally around the nucleus. In contrast, HR1 domain mutations disrupt the conformational dynamics of MFN2 while preserving GTPase function. These variants produce small mitochondria that lack both depolarization and perinuclear accumulation yet ultimately show structural instability [91].

**Figure 2:**
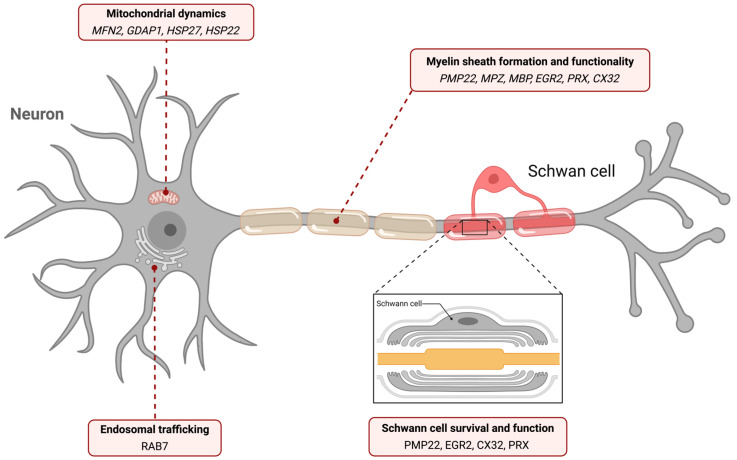
Biological mechanisms of genes associated with hereditary polyneuropathies in neurons and Schwann cells. PMP22: Peripheral myelin protein 22. MPZ: Myelin protein zero, MPB: Myelin basic protein, MFN2: Mitofusin 2, GDAP1: Ganglioside-induced differentiation associated protein 1, HSP27: Heat shock protein family B member 1, HSP22: Heat shock protein family B member 8, EGR2: Early growth response protein 2, PRX: Periaxin, CX32: Connexin 32, TTR: Transthyretin. Created in BioRender. Dermitzakis, I. (2026) https://BioRender.com/nc427zs.

In addition to *PMP22* and *MPZ*, the genes *EGR2* and *PRX* also contribute to the development of DSS. As EGR2 regulates several essential myelin-related genes, such as *MPZ*, *MAG*, and *PMP22*, mutations in *EGR2* disrupt these regulatory interactions, diminishing the transcriptional activation of these targets and ultimately reducing myelin formation [124]. Furthermore, pathogenic variants that impair RNF20/RNF40-mediated regulation prevent Schwann cells from achieving full differentiation, thereby leading to defective myelination [125]. Conversely, certain *PRX* mutations result in excessive myelin production, followed by repeated cycles of demyelination and remyelination. Although the mechanisms controlling myelin sheath thickness are not yet fully understood, they appear to be modulated by axonal signals, as *PRX* expression is regulated through axon–Schwann cell contact [142]. A molecular exploration of the molecular pathways governed by these genes reveals that they are intricately intertwined, reflecting finely balanced regulatory dynamics and raising important questions regarding the extent and nature of their interdependent interactions. The biological pathways related to the aforementioned genes are depicted in Figure 2.

## 4. Treatment Approaches

### 4.1. Current Treatment

At present time, there is no definite cure for HPNs. Only supportive treatment like physical therapy is established, in order to preserve mobility of the upper and lower limbs, while some orthotic devices such as AFO are encouraged for walking difficulties. Medications like gabapentin or amitriptyline for neuropathic pain are used, while surgical interventions are required in cases of severe deformities or functional impairments like per cava [168]. Through recent advances in medicine, more specific medications have been discovered and approved for the treatment of HPNs, although their widespread use is still far from clinical reality.

### 4.2. Research on Conservative Drugs

Over the past decade, significant progress has been made in the research of CMT, leading to the development of various therapeutic approaches aimed primarily at stabilizing the disease, alleviating symptoms, and improving patients’ quality of life. Although molecular therapies represent a highly promising approach for CMT1A, they remain at the preclinical stage [169]. In contrast, more conventional pharmacological strategies have progressed to clinical trials, with some nearing regulatory approval. The most advanced among these is PXT3003, a combinatorial oral formulation consisting of baclofen, naltrexone, and D-sorbitol, currently in Phase III clinical trials [170]. Each of these agents targets *PMP22* overexpression, the underlying pathogenic mechanism in CMT1A. Baclofen, a γ-aminobutyric acid (GABA)-B receptor agonist commonly prescribed for spasticity, downregulates *PMP22* transcription via a cAMP-dependent silencer element in Schwann cells [171]. Naltrexone, an opioid receptor antagonist, is believed to enhance endogenous endorphin release and increase membrane localization of opioid receptors, thereby augmenting the inhibitory effect of endogenous opioids on *PMP22* expression [172]. D-sorbitol, a sugar alcohol used in food and pharmaceutical products, can bind with high affinity to muscarinic receptors, potentially modulating *PMP22* transcription and contributing to protein folding homeostasis [173]. Preclinical studies have demonstrated that PXT3003 reduces *PMP22* expression, promotes normalized Schwann cell differentiation, and improves neuromuscular function [174]. These effects translate into motor and sensory improvement in both upper and lower limbs of patients. However, optimal dosing remains under investigation due to the occurrence of minor gastrointestinal and neurological side effects [175].

### 4.3. Advance on Precision Medicine in HPN Therapies

Recent advances in the treatment of CMT are increasingly grounded in the principles of precision medicine, a rapidly evolving field that tailors therapeutic interventions to the patient’s specific genetic background [176,177]. Approaches such as adeno-associated viral (AAV)-mediated gene therapy, CRISPR/Cas9 genome editing, and antisense oligonucleotides (ASOs) exemplify this paradigm by targeting the underlying genetic causes of disease at a molecular level. These modalities hold promise for personalized interventions, moving beyond symptom management toward disease modification.

#### 4.3.1. CMT1A Therapies

Current preclinical strategies focus on gene silencing techniques aimed at reducing *PMP22* expression, particularly in cases involving the 1.4 Mb duplication, while avoiding excessive knockdown that could lead to HNPP [178]. Highly promising approaches involve the use of AAV vectors with minimal viral genetic content, thereby minimizing immune responses. These vectors enable efficient gene delivery to Schwann cells, motor neurons, and muscle tissue, effectively targeting *Pmp22* mRNA in rat models of CMT1A [61]. Downregulation of *Pmp22* expression enhances myelination, thereby preventing both motor and sensory deficits [179].

A notable example is the therapeutic microRNA miR871, which was encapsulated in an AAV9 vector and administered via lumbar intrathecal injection in a C61-het mouse model expressing both human and mouse *PMP22* [180]. The efficacy of the miR871-AAV9 complex relies on sequence homology between murine *pmp22* and human *PMP22* exons, while its limited off-target effects represent a key advantage. In this study, the treatment cohorts were divided based on the timing of the injection: early treatment at 2 months, late treatment at 6 months, and extended early treatment to evaluate long-term effects. The early treatment group showed full recovery of motor performance in rotarod, grip, and hang tests, as well as normal hind-limb clasping behavior. NCV increased, myelination improved, and inflammation in the peripheral nervous system as well as circulating biomarkers NF-L and Gdf15 decreased. The late treatment group exhibited partial improvement in motor performance and NCV, with a reduction in PMP22 levels and demyelination, but without full restoration to normal levels. Circulating biomarkers showed limited improvement, and peripheral nervous system inflammation was reduced. The extended early treatment group achieved almost complete recovery of functional, morphological, and electrophysiological abnormalities, surpassing the outcomes of the late treatment group [180]. Importantly, motor performance in treated mice improved proportionally to the age at the time of intervention, with early treatment fully reversing the hind limb clasping phenotype, but even late-stage treatment provides partial benefit [181].

Recent preclinical studies in rodents and non-human primates have further optimized vector tropism and delivery strategies. AAV2/9-mediated delivery of shRNA against *Pmp22* has demonstrated high specificity for transducing myelinating Schwann cells along the length of sciatic nerves in non-human primates. Using non-traumatic intra-nerve injection, AAV2/9 transduced up to 95% of Schwann cells in rodents, with significant diffusion several centimeters from the injection site. This approach normalized PMP22 protein levels without affecting mRNA stability, reduced myelin sheath defects, corrected internodal lengths and increased the density of large, myelinated fibers. Functionally, treated animals exhibited long-term preservation of NCV, motor coordination, and sensory perception for at least 12 months [179].

The most noteworthy adverse effect reported was a transient increase in CD20+ and CD3+ immune cell populations in the liver six weeks post-injection; however, this effect was resolved by the four-month time point [180]. Nevertheless, clinical trials differ substantially from preclinical studies and require a meticulously designed approach with minimal side effects. Although AAV9-miR871 treatment did not induce inflammation in peripheral nervous system tissues in murine models, there remains considerable concern that long-term overexpression of miRNAs in humans may interfere with endogenous regulatory mechanisms, potentially leading to toxicity [182].

Another promising approach to reduce *PMP22* expression involves the targeted editing of the TATA-box within the human *PMP22* promoter, a key regulatory element controlling transcriptional activity. In a CMT1A mouse model, localized intraneural delivery of CRISPR/Cas9 constructs designed to disrupt this promoter region led to ∼40–45% reduction in human *PMP22* mRNA without detectable off-target effects, preserving a favorable safety profile. Histological analyses revealed decreased unmyelinated axons, reduced “onion bulb” formations, increased myelin thickness, and higher numbers of large-diameter axons. Functionally, treated mice showed increased motor NCV, along with greater gastrocnemius muscle weight, indicating enhanced muscle innervation [183]. Notably, the same intervention showed partial reversal of demyelination and functional improvement even when applied after the onset of neuropathic symptoms. Targeting the TATA box enables specific downregulation of *PMP22* transcription in Schwann cells without excessive silencing, minimizing the risk of HNPP, and demonstrates the potential of single-dose, non-viral CRISPR/Cas9 therapy as a durable intervention for CMT1A and potentially other gene duplication disorders [181].

Antisense oligonucleotides (ASOs) represent a promising gene therapy approach for HPNs, particularly for CMT1A. ASOs are short, synthetic, single-stranded nucleic acids, designed to bind specifically to target RNA sequences through complementary base pairing. By doing so, they can modulate gene expression through various mechanisms—such as promoting RNA degradation and blocking translation [184]. This advanced strategy aims also at suppression of *PMP22* expression and is currently at preclinical trials in mice. Weekly subcutaneous injection of 25, 50 or 100 mg/kg ASOs targeting the open reading frame of *PMP22* and *Pmp22* in 5-week-old C22 mice and 6-week-old heterozygote CMT1A rats resulted in dose-depended *PMP22*/*Pmp22* silencing as well as improved myelination and electrophysiological performance of the models for up to 12 weeks post-injection. As ASO treatment alters the transcriptional profile of Schwann cells in favor of myelination, this method seems to decrease *PMP22* mRNA levels and improve behavioural, electrophysiological, and pathological features of neuropathy [185]. Research suggests that ASOs result in the myelination of demyelinated axons, contributing to faster conduction and even more conducting myelinated motor axons. Remarkably, ASO treatment initiated after disease onset in CMT1A rats reversed the severity of the neuropathy [186]. These promising results raise the question of whether repeated ASO injections can lead to sustained improvement of CMT1A phenotype. In order to address this question, it is important to determine the half-life of the ASOs in the body [178]. Additionally, research has shown that the decrease of *PMP22* mRNA in skin biopsies from rats treated with ASO is a good biomarker for assessing target engagement in response to ASO treatment [185]. These findings provide evidence in favor of using ASOs as a possible treatment for CMT1A and clarify potential biomarkers of illness and target engagement for use in upcoming clinical studies [185].

Although ASOs have shown promise in preclinical models of inherited polyneuropathies, several challenges must be addressed before clinical application. A major limitation is their poor permeability across the blood–nerve barrier, necessitating high systemic doses that may lead to toxicity. Recent research efforts have focused on enhancing targeted delivery of ASOs to Schwann cells by employing nanoparticle-based delivery systems. For instance, calcium phosphate–lipid nanoparticles (CaP-lipid NPs), have been developed to improve the delivery of ASOs to motor neurons, demonstrating effective gene silencing and reduced toxicity in animal models [187]. These nanoparticle carriers facilitate endocytosis and promote endolysosomal escape, thereby enhancing the intracellular availability of ASOs. Such strategies aim to increase therapeutic efficacy while minimizing the required dosage and associated side effects [188].

#### 4.3.2. CMT2A Therapies

CMT2A, resulting from mutations in *MFN2*, is also in the centre of interest. Researchers found that SARM1, a key protein in axonal degeneration, worsens mitochondrial pathology when activated by dysfunctional mitochondria. In a rat model carrying the dominant *MFN2* H361Y mutation, researchers generated double-mutant animals lacking SARM1 (Sarm1^−/−^) and found that deletion of SARM1 rescued axonal degeneration, neuromuscular junction abnormalities, muscle atrophy, and the progressive ‘dying-back’ neuropathy otherwise caused by mutant *MFN2* [189]. Remarkably, despite the presence of mutant MFN2 protein, SARM1 deletion also dramatically suppressed many mitochondrial defects, including reduced mitochondrial number and abnormal size, suggesting a positive feedback loop wherein dysfunctional mitochondria activate SARM1, which in turn worsens mitochondrial pathology. This suggests that SARM1 inhibition could represent a promising therapeutic approach for CMT2A and other neurodegenerative diseases linked to mitochondrial dysfunction [189].

Small-molecule mitofusin agonists target the mitochondrial fusion machinery by allosterically activating *MFN2*. In the context of CMT2A, mutations in *MFN2* undermine mitochondrial fusion, resulting in mitochondrial fragmentation, impaired axonal mitochondrial transport, and progressive axonal degeneration. By stabilizing MFN2 in its “open/active” conformation, mimicking key side-chains (Val372, Met376, His380) of the HR1-HR2 interface, mitofusin agonists promote mitochondrial tethering and fusion, thereby restoring mitochondrial morphology and transport in affected neurons [90]. Early phenylhexanamide compounds, such as compound 2, showed potent mitochondrial fusion activity but had very short in vivo half-lives (~1 h), providing only transient activation. To improve stability and CNS exposure, cycloalkyl linkers, particularly a cyclopropyl group in “compound 5”, were introduced, yielding the trans-enantiomer (5B) as the fully active form [190]. Preclinical studies in murine CMT2A models demonstrated that 5B increased mitochondrial motility, improved neuromuscular function, and sustained mitofusin activation for over 24 h, offering superior pharmacodynamic effects compared to earlier compounds. In preclinical models of CMT2A, mitofusin agonists produced encouraging results. Mitochondrial fusion and motility defects in MFN2-mutant neurons were ameliorated; mitochondrial transport along axons improved in a dose-dependent manner; daily or twice-daily administrations reversed neuromuscular degeneration, and benefits were evident even in older even in older animals [91].

#### 4.3.3. Neurotrophic Factors for Treatment; HGF, NT-3, BDNF

A novel nonviral plasmid DNA therapy, VM202, which encodes human hepatocyte growth factor (HGF), is currently being explored as a potential treatment for CMT1A. Originally identified as a potent mitogen for hepatocytes, HGF is now recognized for its broader biological roles, including neurotrophic, angiogenic, and antifibrotic effects [191]. These effects are mediated via the c-Met receptor, which is expressed in several cell types such as Schwann cells, peripheral neurons, and muscle stem cells [192]. Studies have shown that HGF supports peripheral sensory and motor neurons by promoting axonal growth and enhancing neuronal survival. Additionally, it mitigates neurogenic muscle atrophy by increasing the expression of miR-206—a microRNA involved in muscle differentiation through the regulation of myogenic regulatory factors [193]. Building on these findings, researchers administered VM202 via intramuscular injections in both legs of CMT1A patients [189]. The treatment was well tolerated, with peripheral edema being one of the few reported side effects. Notably, the transient nature of HGF expression from VM202 reduced the risk of antibody formation, further supporting its safety profile [194].

Similarly, NT-3 is a neurotrophic factor, crucial for Schwann cells’ autocrine survival and regeneration [195]. In recent preclinical and early clinical studies, NT-3 was packaged in AAV1 and delivered via intramuscular injection in both animal models and patients with CMT1. This approach resulted in robust axonal regeneration and improved myelination, with beneficial effects lasting up to 40 weeks post-injection [74]. Muscle tissue was selected as the AAV target because it can function as a secretory organ for systemic and long-term NT-3 distribution. AAVs are also being investigated in preclinical studies for the treatment of CMTX1, where the gene encoding NT-3 is delivered via intramuscular injection of a self-complementary AAV1 vector [196]. Long-term results showed a measurable increase in NT-3 production six months post-treatment, ensuring the sustained functionality of Schwann cells. Over this period, myelination improved, the frequency of onion bulb formations decreased, and the occurrence of active demyelination and naked axons was significantly reduced [197]. This treatment approach not only targets the defective protein but also promotes the overall regeneration of Schwann cells, raising hope for its potential application in other polyneuropathies. Furthermore, NT-3 therapy increased the density of myelinated neurons and Schwann cells, improved neurofilament cytoskeleton integrity and neurophysiological parameters and enhanced motor performance, with higher improvements reported after prolonged treatment [198]. Interestingly, NT-3 has also shown efficacy in mouse models of an additional CMT subtype, CMT2D, supporting its potential as a broad-spectrum therapeutic factor in inherited neuropathies [199].

A third neurotrophic factor, brain-derived neurotrophic factor (BDNF), has emerged as a promising therapeutic candidate for certain genetic forms of CMT2D. BDNF plays a critical role in neural development and is known to modulate synaptic plasticity, thereby influencing circuit formation and cognitive function [200]. To achieve sustained local expression, the vector AAV8-tMCK-BDNF was utilized, enabling prolonged BDNF production in skeletal muscle. However, therapeutic effects were confined to the injected muscle, with no observable benefit in contralateral or non-targeted muscles [198]. These findings underscore the need for optimized delivery strategies to achieve systemic or broader neuromuscular efficacy, rather than localized improvements alone.

#### 4.3.4. hATTR Therapies

In recent years, therapeutic innovation has focused on early-stage disease, resulting in the approval of two first-in-class RNA-targeting therapies: patisiran and inotersen. While these agents offer significant benefit to patients with stage 1 or 2 polyneuropathy, there are currently no approved treatments for patients with advanced disease [201].

Patisiran and inotersen differ in their mechanisms of action but share the common goal of reducing hepatic production of TTR, thereby mitigating amyloid deposition in peripheral nerves. Patisiran is a double-stranded small interfering RNA (siRNA) encapsulated in lipid nanoparticles. It binds to a conserved sequence in the 3′ untranslated region (3′UTR) of both mutant and wild-type *TTR* mRNA, leading to mRNA degradation via the RNA interference (RNAi) pathway. This results in a significant reduction of circulating TTR levels, preventing amyloid fibril formation and limiting organ damage [202]. Inotersen in contrast, is a second-generation 2′-O-(2-methoxyethyl) ASO that binds to *TTR* mRNA, inducing degradation through the RNase H1 pathway. This similarly decreases hepatic production of both mutant and wild-type *TTR*, slowing disease progression [187].

In preclinical models, RNAi-mediated knockdown of *TTR* expression led to significant reductions in amyloid burden in peripheral tissues, with the degree of regression correlating to serum TTR levels [141]. Clinical trials further validated these findings: patisiran demonstrated sustained stabilization of neuropathy impairment scores over 24 months, while inotersen showed comparable efficacy over 18 months of treatment [187,203].

Beyond efficacy, the clinical administration and safety profiles of these therapies differ, which has implications for patient management. Patisiran is given intravenously once every three weeks, whereas inotersen is administered subcutaneously on a weekly basis. Moreover, patisiran has been associated with a more favorable safety profile, with lower incidences of thrombocytopenia and renal adverse events compared to inotersen, which requires regular monitoring of platelet counts and renal function [187,203].

In addition to controlled trials, real-world data continue to support the effectiveness of RNA-targeted therapies in broader patient populations, confirming their clinical value outside the trial setting. Furthermore, the therapeutic landscape for hATTR amyloidosis is rapidly evolving, with novel agents currently in development [204]. These include oral TTR stabilizers such as acoramidis, and potentially curative CRISPR-Cas9-based gene editing therapies like NTLA-2001, which have shown promising early results in reducing serum TTR levels in human trials [205].

Next generation subcutaneous RNA therapies, such as a hepatocyte-directed antisense oligonucleotide, eplontersen, and a chemically stabilized siRNA, vutrisiran, represent the latest generation of RNA-targeted therapeutics designed to improve convenience, potency, and long-term tolerability. Eplontersen is a subcutaneously administered N-acetylgalactosamine (GalNAc-conjugate) ASO that enables efficient hepatocyte uptake and potent *TTR* mRNA degradation via the RNase H1 pathway [206]. Research results showed durable (>80%) reductions in serum TTR concentration, stabilization or improvement in neuropathy scores, and significant gains in quality-of-life indices, leading to recent regulatory approvals in several regions. Likewise, vutrisiran, evaluated in the pivotal *HELIOS-A* and *HELIOS-B* trials, produced sustained *TTR* knockdown with infrequent subcutaneous dosing, achieving efficacy comparable to patisiran while demonstrating improved convenience and safety. In patients with ATTR cardiomyopathy, vutrisiran treatment was associated with reduced mortality and cardiovascular morbidity [207]. Both agents show favorable pharmacodynamic profiles and most importantly, these developments illustrate that targeted RNA knockdown can produce durable biomarker suppression and translate into clinically meaningful neurological and systemic benefit.

#### 4.3.5. Advances in Precision Diagnosis

Next-generation sequencing (NGS) encompassing targeted panels, whole-exome, and whole-genome sequencing, has significantly increased the detection of causative variants, including single-nucleotide changes, copy number variations, and deep intronic mutations [5]. RNA sequencing (RNA-seq) complements DNA-based testing by revealing splicing defects, allele-specific expression, and transcript-level dysregulation that contribute to disease phenotypes. Long-read transcriptomic approaches further enhance the ability to resolve complex isoforms, clarifying pathogenic mechanisms and allowing the reclassification of previously uncertain variants. Functional validation in patient-derived cells provides essential evidence for variant pathogenicity, confirming mechanistic links and supporting gene-targeted therapies [208]. Through these diagnostic methods, genotype connects to molecular mechanism and, therefore, to therapeutic potential, providing molecular biomarkers to monitor disease progression and treatment response.

#### 4.3.6. General Considerations and Challenges

Although advance in gene therapy for HPNs is significant, there are certain obstacles that continue to limit clinical translation. First, efficient delivery of therapeutic vectors to peripheral nerves remains a significant challenge. The blood–nerve barrier restricts vector penetration, while the long axonal architecture of human peripheral nerves complicates widespread transduction [181]. As described previously, recent studies have explored strategies such as intrathecal or intraneural injection, in order to enhance Schwann cell and axonal targeting [180]. Other strategies include AAV capsid engineering and retrograde transport optimization, raising hope for less mistargeting and more efficient outcome. Secondly, immune responses to AAV vectors pose substantial safety and efficacy concerns. Pre-existing neutralizing antibodies and activation of the adaptive immune system can hinder repeated vector administration and may lead to toxicity, inflammatory responses, and neuronal loss in specific brain regions, especially when the constructs are overexpressed. The safety of these approaches depends on factors like AAV dose, the type of RNAi construct, and promoter choice [182]. Current efforts focus on minimizing immunogenicity through the development of novel low-immunity capsids, transient immunosuppression protocols, or non-viral delivery systems [209]. Finally, the difficulty in reproducing late-onset, slowly progressive neuropathy in experimental models represents another major barrier. Most rodent models exhibit rapid disease onset and short axonal length, making it challenging to evaluate long-term safety, efficacy, and durability of gene therapies. Consequently, non-human primate and large-animal models are increasingly being utilized to better mimic human peripheral nerve pathology and to assess the chronic effects of AAV-mediated gene modulation [210].

## 5. Conclusions and Future Directions

This review underscores the pivotal role of genetic mutations in the development of HPNs, highlighting the significant complexity and variability of these disorders. Insights into the genetic mechanisms have revealed the contributions of genes such as *PMP22, MPZ, MFN2, TTR, EGR2, PRX*, and *CX32* to the pathophysiology of these conditions, further illustrating their intricate and diverse nature. Additionally, other genes appear to influence the polyneuropathy phenotype, although their molecular underpinnings remain incompletely understood. With more than 140 associated genes identified, the molecular complexity poses substantial challenges for accurate diagnosis and, consequently, effective treatment.

By synthesising current knowledge on the known genes and their associated mutations, this review aims to provide an overview of the genetic background, pathophysiology, and resulting phenotypes of HPNs. It further outlines the close relationships and shared mechanisms among certain neuropathies and summarizes both established therapeutic approaches and emerging treatment strategies under investigation. Through the analysis of these aspects, the review intends to serve as a useful and comprehensible resource for researchers and clinicians aiming at the development of innovative therapeutic approaches. Although other reviews on this topic exist, this work seeks to offer an integrative perspective by bringing together genetic, pathophysiological, clinical, and therapeutic insights within a single, cohesive framework.

Despite the absence of curative treatments for HPNs, remarkable progress has been made in both understanding the genetic basis and developing therapies for these disorders. Precision medicine approaches including gene silencing technologies like ASOs, genome editing with CRISPR/Cas9, and gene replacement via AAV vectors offer promising ways to directly address the underlying mutations and adapt treatment to each patient’s specific genetic profile. Future research should focus on further clarifying the role of key genes, such as *PMP22, MPZ, MFN2, TTR, EGR2, PRX,* and *CX32*, improving patient-derived and animal models to better study disease mechanisms, and optimizing delivery methods to maximize safety and efficacy. Future research should further investigate dosage-dependent effects and the influence of environmental exposures, as both represent complex and deeply layered determinants of the pathophysiology of polyneuropathy that offer promising avenues for meaningful scientific advancement. For example, the clinical variability observed among patients carrying the same *PMP22* duplication suggests that gene dosage interacts with additional biological factors, such as modifier genes, epigenetic regulation, and metabolic state. Environmental contributors, such as chronic alcohol consumption, exposure to neurotoxic agents, nutritional deficiencies and metabolic dysregulation, may further modulate disease onset, severity, or rate of progression, highlighting the need for integrated genetic–environmental analyses.

The influence of copy number variants in *PMP22* and the other dosage-sensitive genes, should also be a major focus, with future research assessing not only their direct impact on myelin homeostasis but also how coexisting variants may modulate disease severity or generate atypical clinical presentations. Additionally, metabolic and inflammatory factors may act as key secondary modulators affecting the manifestation and progression of HPNs. Chronic systemic inflammation, impaired lipid metabolism, mitochondrial dysfunction, and oxidative stress have all been implicated in accelerating neural injury in genetically susceptible individuals. The examination of those aspects could reveal potential targets for personalized therapeutic interventions. Combining clinical data with precision strategies and other determinants will be important for tracking gene progression and supporting the development of effective therapies and personalised care. Continued investment in delivery systems, such as nanoparticle-based carriers and tissue-specific targeting, will play a key role in this achievement.

## Figures and Tables

**Table 1:** Genetic alterations and pathophysiology mechanisms in *PMP22*-associated HPNs.

Genetic Alterations	Pathophysiology Mechanism	Disease	Ref.
Duplication of 17p11.2–p12	Boost for P2RX7 expression → increase in calcium levels in mature Schwann cells → segmental demyelination	CMT1, RLS	[6,12,13,14]
Asp37Val substitution in the first extracellular domain	Overexpression of *PMP22* → dysregulation of myelin proteins expression → suppression of the enzymes that catalyse cholesterol production → myelin thickness and shorter internodes	CMT1A	[15,16,17]
C > T substitution at c.402 (exon 4)	-	CMT1	[18]
G > A substitution at c.178	-	CMT1	[19]
Deletion of exon 4	-	CMT1	[19]
p. His12Pro variation	-	CMT1	[19]
p. Thr118Me	-	CMT1A in homozygosity; HNPP in heterozygosity	[18]
G > A substitution at c.202 (exon 3)	Decreased amount of PMP22 → insufficient for myelin formation → axons vulnerable to mechanical damage	HNPP	[11,20,21]
Deletion of 17p11.2–p12	-	HNPP	[11,19]
Ser to Leu substitution	Supernumerary of Schwann cells, causing onion bulb formation and thickening of the myelin sheath	DSS	[22,23]
C > A substitution at c.85	-	DSS	[24]
Met69Lys	-	DSS	[25,26]
Leu16Pro	-	DSS	[25,27]
Leu70Arg	-	DSS	[25,27]
Ser72Leu	-	DSS	[27]
T > C substitution at c.374 (exon 3)	-	CHN	[28,29]

The symbol (-) indicates that the pathophysiological mechanism associated with this genetic alteration has not been clearly elucidated. CHN: Congenital hypomyelinating neuropathy, CMT: Charcot–Marie–Tooth disease, DSS: Dejerine–Sottas syndrome, HNPP: Hereditary neuropathy with liability to pressure palsies, HPNs: Hereditary polyneuropathies, PMP22: Peripheral myelin protein 22, P2RX7: Purinergic receptor, Ref.: Reference, RLS: Roussy–Levy syndrome.

**Table 2:** Genetic alterations and pathophysiology mechanisms in *MPZ*-associated HPNs.

Genetic Alterations	Pathophysiology Mechanism	Disease	Ref.
C > T substitution at c.277 (exon 2)	ER retention, activation of the UPR, mis-glycosylation and disruption of myelin compaction, mistrafficking of mutant MPZ to the myelin sheath	CMT1B	[62,63]
G > A substitution at c.444 (exon 3)	-	CMT1B	[30]
replacement of Tyr181by a termination codon	-	CMT1B	[30]
replacement of Tyr154 by a termination codon	-	CMT1B	[30]
Arg98Cys	-	CMT1B	[64]
Ser63 deletion	-	CMT1B	[64]
Lys130Arg	-	DSS	[25,65]
Thr34Ile	-	DSS	[25,65]
Ser54Cys	-	DSS	[25,65]
Ile135Leu	-	DSS	[25,65]
Arg138Cys	-	DSS	[25,65]
Ile32Phe	-	DSS	[25,65]
Cys63Ser	-	DSS	[66]
Arg168Glyc	-	DSS	[66]
Arg69Cys	-	DSS	[46]
Ile1Met	-	DSS	[58]
Ile33Phe	-	DSS	[58]
Ser34Cys	-	DSS	[58]
Tyr53Cys	-	DSS	[58]
Lys101Arg	-	DSS	[58]
Ile106Thr	-	DSS	[58]
Deletion of Phe64	-	DSS in homozygosity; CMT in heterozygosity	[67]
Frameshift mutations resulting in amino acid change after Leu-145 in the transmembrane domain	Defective myelin compaction	DSS	[46,68]
Frameshift mutation that causes amino acid change after Gly-74 in the extracellular domain	-	DSS	[46,68]
Replacement of Gln-186 by termination codon	Escape of nonsense-mediated mRNA decay → truncated proteins that act in a dominant-negative way, interfering with normal MPZ function	CHN	[63,69]
C > A substitution at c.727 (exon 3)	Affected myelin structure	RLS	[49,51]
C > T substitution at c.428	-	RLS	[68,70]
Asn102Lys	-	RLS	[58]

The symbol (-) indicates that the pathophysiological mechanism associated with this genetic alteration has not been clearly elucidated. CHN: Congenital hypomyelinating neuropathy, CMT: Charcot–Marie–Tooth disease, DSS: Dejerine–Sottas syndrome, ER: Endoplasmic reticulum, HPNs: Hereditary polyneuropathies, MPZ: Myelin protein zero, PMP22: Peripheral myelin protein 22, Ref.: Reference, RLS: Roussy–Levy syndrome, UPR: Unfolded protein response.

**Table 3:** Overview of hereditary polyneuropathies.

Disease	Phenotype	Lesions	Genes	Mechanism	Ref.
CMT1A	Symmetrical progressive muscle weakness and atrophy, limb deformities, loss of sensation, diminished/ absent tendon reflexes	Slow NCVs, axonal loss	*PMP22* (overexpression)	Dysregulation of myelin proteins → lack of cholesterol → lower myelin thickness and shorter internodes Increase in calcium levels by P2RX7 → segmental demyelination	[13,14,16,17,18,19,20,24]
HNPP	Asymptomatic, gradual muscle weakness, located sensory loss, limping and refusal to walk, recurrent and self-limited nerve paralysis	Distinctive localized thickening of the myelin sheaths (tomacula), variable axonal loss, slow NCVs	*PMP22* (loss of function)	Decreased PMP22 → increased permeability of myelin sheath → peripheral nerves susceptible to pressure induced injuries	[11,34,35,37,38,39]
DSS/CHN	Similar to CMT1A, but earlier appearance, hypesthesia, hypotonia, areflexia and thoracolumbar kyphoscoliosis, mild ptosis and limitations of eye movements, distal muscle weakness, joint contractures in the severe form, greater morbidity	Hypomyelination and basal laminae onion bulbs, slow NCVs	*PMP22*	Supernumerary of Schwann cells → myelin formation deficiency → onion bulb formation and thickening of myelin sheath, demyelination, remyelination	[22,23,26,29,43,44,45]
			*MPZ*	Defective myelin compaction, accumulation of misfolded MPZ in ER → Schwann cells stress	[63,68,69]
			*EGR2*	Reduced transcriptional activation of myelin-related genes, failure of Schwann cells full differentiation → defective myelination	[119,125]
			*PRX*	Enzymatic defects, unstable cytoskeleton and myelin of Schwann cells → inability of rapid conduction of nerve signaling	[143,144]
RLS	Tremor, ataxia, muscular atrophy, bilateral pes cavus, loss of vibratory and position sense in upper and lower limbs, wide-based and ataxic gait and areflexia, mild scoliosis of the lower thoracic spine, high life expectancy	Hypertrophic and demyelinating lesions, slow NCVs, onion bulbs	*PMP22*	Reduction of nerve conduction demyelination, presence of onion bulbs, shared mechanisms to CMT1A	[47,49,51,52]
			*MPZ*	Dysfunctional myelin in the ER	[58,70]
CMT1B	Muscular weakness, foot abnormalities, sensory loss and discomfort, absent tendon reflexes, pes cavus and decreased sensation in distal extremities, bilateral facial weakness and absence of vibratory sensation in both legs	Slow NCVs	*MPZ* (gain of function and rarely loss of function)	Mutant MPZ remains in the ER rather than being transported to the cell membrane, activation of the UPR, mis-glycosylation and disruption of myelin compaction, mistrafficking of mutant MPZ to the myelin sheath, all leading to demyelination	[62,68,74,75,76]
CMT2A	Muscle weakness, greater severity of disability than CMT1, difficulty in walking, due to deformities of the lower limbs, tendon areflexia, leg muscle cramps, pes cavus, distal paralysis, finger tremor	Slow NCVs, decreased amplitudes of evoked motor and sensory nerve responses, variable loss of myelinated fibers, small onion bulbs	*MFN2*	Axonopathy by impairing energy production along the axon, mitochondrial fragmentation without depolarization or perinuclear aggregation and block of final fusion step, unfolded protein	[82,87,89,90,91,93,94]
hTTR	Lower limb paresthesia, cardiomyopathy, gastrointestinal symptoms, postural hypotension, bladder dysfunction, paresthesia, burning and extending pain in the feet, dysesthesias, and dysautonomia	Slow NCVs, prolonged distal motor latencies	*TTR*	Axonal damage, decrease in repair after damage	[109,111,112,167]
CMT4F	Delay in development, distal muscle atrophy, mild kyphoscoliosis	Onion bulbs formation	*PRX*	Absence of a large repeat-rich domain, excessive myelin production de-remyelination cycles	[140,144,145]
CMT1X	Muscle weakness, sensory loss and atrophy with greater severity in males than females	Not well defined	*CX32*	Dysregulation of immune-responding genes, obstruction of gap junction formation → failure in maintenance of myelin integrity → axonal	[152,156]

## Data Availability

No new data were created or analyzed in this study.

## Abbreviations

The following abbreviations are used in this manuscript:

AAV	Adeno-associated viral
AD	Alzheimer’s disease
AFO	Ankle foot orthoses
ARHGEF10	Rho guanine nucleotide exchange factor 10
ASOs	Antisense oligonucleotides
BDNF	Brain-derived neurotrophic factor
CDRT15	CMT1A duplicated region transcript 15
CHN	Congenital hypomyelinating neuropathy
CMT	Charcot–Marie–Tooth disease
CNS	Central nervous system
CSF	Cerebrospinal fluid
Cx32	Connexin 32
DBD	DNA binding domain
DCTN1	Dynactin subunit 1
DSS	Dejerine–Sottas syndrome
EGR2	Early growth response protein 2
ER	Endoplasmic reticulum
GARS	Glycyl-tRNA synthetase
GDAP1	Ganglioside induced differentiation associated protein 1
HGF	Human hepatocyte growth factor
HNPP	Hereditary neuropathy with liability to pressure palsies
HPNs	Hereditary polyneuropathies
HR	Heptad repeat region
HSP22	Heat shock protein family B member 8
HSP27	Heat shock protein family B member 1
HS3ST3B1	Heparan sulfate-glucosamine 3-sulfotransferase 3B1
hTTR	Hereditary transthyretin amyloidosis
KIF1B	Kinesin family member 1B
KO	Knockout
MAG	Myelin-associated glycoprotein
MBP	Myelin basic protein
MFN2	Mitofusin 2
MPZ	Myelin protein zero
NCV	Nerve conduction velocity
NEFL	Neurofilament light chain
NGFI-A	Nerve growth factor induced-A
NGFR	Nerve growth factor receptor
NGS	Next generation sequencing
ΝΤ-3	Neurotrophin-3
Pmcao	Permanent middle cerebral artery distal occlusion
PMP22	Peripheral myelin protein 22
PLS	Potocki–Lupski syndrome
PNS	Peripheral nervous system
PRX	Periaxin
RAB7	Member of the RAS oncogene family
RLS	Roussy–Levy syndrome
RNA-seq	RNA sequencing
SARM 1	Sterile alpha and TIR motif containing 1
SMS	Smith–Magenis syndrome
SNHI	Progressive sensorineural hearing impairment
TEKT4	Tektin 4
TTR	Transthyretin
UPR	Unfolded protein response
